# The role of the vesicular monoamine transporter 2 in the inhibitory effect of tetrabenazine and valbenazine compared to reserpine on the vesicular release of monoamine transmitters

**DOI:** 10.3389/fncel.2025.1648613

**Published:** 2025-11-07

**Authors:** Fruzsina Maácz, Erika Gyöngyi Bán, Attila Brassai, Beáta Sperlágh, E. Sylvester Vizi

**Affiliations:** 1 Laboratory of Molecular Pharmacology, HUN-REN Institute of Experimental Medicine, Budapest, Hungary; 2 Szentágothai János Neuroscience Division, Semmelweis University Doctoral School, Budapest, Hungary; 3 Department of ME1, Faculty of Medicine in English, “George Emil Palade” University of Medicine, Pharmacy, Science and Technology of Târgu-Mureș, Marosvásárhely, Romania; 4 Institute of Pharmacology and Pharmacotherapy, Semmelweis University, Budapest, Hungary

**Keywords:** vesicular monoamine transporter, noradrenaline, serotonin, reserpine, tetrabenazine, valbenazine, vesicular storage capacity, exocytotic release

## Abstract

**Background:**

Vesicular monoamine transporter 2 (VMAT-2) plays a vital role in packaging cytosolic monoamine transmitters into axon terminal vesicles, which can be released in response to action potentials. Reserpine (RSP), a classical irreversible inhibitor of the monoamine transporter, is an alkaloid used as an antihypertensive drug. However, its use in medicine was very short-lived because of side effects (depression, Parkinsonism). Tetrabenazine (TBZ) and valbenazine (VBZ), biochemically non-competitive and reversible VMAT-2 inhibitors, are both used in the treatment of Tardive Dyskinesia (TD). The aim of this study was to directly compare the effects of RSP, TBZ, and VBZ on vesicular storage and exocytotic release of monoamines in hippocampal slices, and to clarify whether their actions differ in terms of reversibility and persistence. Our work addresses the biological question of how these clinically relevant VMAT-2 inhibitors modulate monoaminergic neurotransmission at the synaptic level.

**Materials and methods:**

Vesicular storage capacity and release of [^3^H] noradrenaline ([^3^H] NA), [^3^H] serotonin ([^3^H] 5-HT), and [^3^H] acetylcholine ([^3^H] ACh) were studied in mouse hippocampus *ex vivo* slice preparations using electrical field stimulation.

**Results:**

In this study, for the first time, direct neurochemical evidence was obtained that RSP reduces the vesicular storage capacity and the exocytotic release of [^3^H] NA and [^3^H] 5-HT evoked by axonal stimulation from the *ex vivo* hippocampal slice preparations and failed to influence the plasma membrane uptake of monoamines and exocytotic release of [^3^H] ACh. The inhibitory effect of RSP on vesicular release, neurochemically proven to be irreversible, was not accompanied by a recovery in VMAT-2 enzyme activity, as observed in biochemical studies. TBZ and VBZ are compared to RSP in that they also inhibit the vesicular release of neurotransmitters and storage capacity; however, their activity is less effective and is of much shorter duration, leaving some time for vesicle refilling.

**Discussion:**

The difference observed between the two types of VMAT-2 inhibitors might give some explanation of why, in response to TBZ or VBZ treatment, the occurrence of depression or Parkinsonism as side effects is seen very rarely or not at all, and in the case of RSP, it is relatively frequent.

## 1 Introduction

During chemical neurotransmission evoked by axonal activity, the transmitter stored in a readily releasable pool of vesicles must be replenished by a continuous supply (Brachtendorf et al., 2015), and the vesicles should be refilled with transmitters from the cytoplasm using a transporter located in the membrane (Eiden et al., 2004; Bravo et al., 2005). Vesicular monoamine transporter type 2 (VMAT-2) is responsible for packaging monoamine neurotransmitters in neuronal vesicles at axon terminals by importing one monoamine neurotransmitter, such as noradrenaline (NA), dopamine (DA), and serotonin (5-HT), in exchange for two cytoplasmic protons via an ATP-dependent mechanism (Johnson et al., 1981).

VMAT-2 is a pharmacological target in drug development (Zhang et al., 2024) for the treatment of psychiatric and neurodegenerative disorders. Its selective non-competitive inhibitors, such as tetrabenazine (TBZ) and valbenazine (VBZ), have already been therapeutically used in Huntington’s disorders and tardive dyskinesia (TD) evoked by second- (Caroff et al., 2018) and third-generation antipsychotic medication. Recent observation has shown that VMAT-2 is also involved in attention deficit hyperactivity disorder (ADHD), which is characterized by changes in executive function and cognitive deficits (Iv et al., 2024), and, accordingly, TBZ was applied for their treatment recently (Porta et al., 2008).

In addition, reserpine (RSP), an irreversible competitive inhibitor of VMAT-2 and VMAT-1 enzymes (Henry and Scherman, 1989), had been used to treat hypertension, and shortly after its introduction, it was withdrawn due to its frequent side effects (depression and parkinsonism) occurring during its application in medical practice. According to the monoamine theory of depression (Carlsson, 1965), this disorder is linked to impaired noradrenergic and serotonergic neurotransmission in the brain. Clinical observations also supported this idea: patients treated with RSP for hypertension often developed depressive symptoms (Strawbridge et al., 2023). Early studies further showed that RSP reduces 5-HT release (Brodie et al., 1956). Nevertheless, aside from the effects of RSP on monoamine levels in the brain (Carlsson, 1978), there is a lack of information on how RSP affects vesicular storage capacity and the axonal activity-related exocytotic release of monoamine transmitters, both of which play a critical role in chemical transmission. In this study, the effects of RSP, compared with those of TBZ and VBZ, were studied on the hippocampus, which plays a vital role in functional interaction with the prefrontal cortex in memory and learning (Vizi and Kiss, 1998) and is accordingly involved in the pathology of depression. In this study, neurochemical evidence was obtained for the first time that RSP impairs noradrenergic and serotonergic neurotransmission due to the long-lasting irreversible inhibition of vesicular storage capacity and, subsequently, the exocytotic release of neurotransmitters NA and 5-HT without influencing ACh release. These effects differ from those produced by TBZ or VBZ in that the non-competitive antagonists exert their effects transiently and surmountable.

## 2 Materials and methods

### 2.1 Animals

In this study, 114 male WT Crl: CD1(ICR) (RRID: IMSR_CRL:022, 5–8 weeks old, weighing 25–33 g were used). WT mice were bred and genotyped at the Medical Gene Technology Unit of the Institute of Experimental Medicine (Budapest, Hungary). Only male mice were used to eliminate the potential effects of the oestrous cycle on animal behavior. Animals were maintained on a 12:12 light–dark cycle in a temperature (23 ± 2 °C) and humidity-controlled room (60 ± 10%), with free access to food (ssniff® Souris-Elevage E, 10 mm pellet, Cat# S8189-S096; ssniff Spezialdiäten GmbH, Soest, Germany) and water. Before the experiments, 4–6 adult littermate mice were kept in standard mouse cages with corncob bedding. To enrich the environment, cardboard bedding material and tubes were placed in each cage. The experimental procedures described in this manuscript have been approved by the Semmelweis University Regional Committee (No. 116/2015) and the local Institutional Animal Care Committee of the IEM HAS (PE/EA/00513-6/2025) and followed the guidelines of the Hungarian Act of Animal Care and Experimentation guidelines (40/2013, II.14), which follows the Directive 2010/63/EU and the recommendations of the Committee on Animals in Research (FENS). Animals were treated humanely, and all effort was made to minimize animal suffering and reduce the number of animals used in experiments.

The sample size was calculated based on a pilot study of [^3^H] NA release after the first stimulation between control and in the presence of 1 μM RSP investigated groups. We calculated the number of animals required per group using G*Power 3.1.9.7 software (RRID: SCR_01372) (Student’s *t*-test: *a priori*: compute required sample size; tail(s): two, power: 0.7; α error probability: 0.05; effect size: 1.367; total sample size: 12).

Animal experiments were reported under ARRIVE 2.0 guidelines. The exact number of mice in each experimental group is given in the legend of the corresponding figures. The mice were randomly assigned to experimental groups, and the investigators were blinded to their experimental status.

### 2.2 Materials

The following chemicals were used for the release experiments: levo-[7-^3^H] norepinephrine (specific activity, 11.8 Ci·mmol^−1^) and 5-hydroxytryptamine-[3H]-trifluoromethylacetate (specific activity, 80 Ci·mmol^−1^) and [^3^H] choline chloride (specific activity, 80 Ci/mmol), purchased from American Radiolabeled Chemicals, Inc. (St. Louis, MO, United States). Hemicholinium-3 (Cat# H108) was obtained from Sigma-Aldrich (St. Louis, MO, United States). The irreversible VMAT-2 inhibitor RSP [(IUPAC name: 3β,16β,17α,18β,20α)-11,17-Dimethoxy-18-[(3,4,5 trimethoxybenzoyl)oxy]yohimban-16-carboxylic acid methyl ester, Cat# 2742] and the reversible VMAT-2 inhibitor TBZ (IUPAC name: 9,10-dimethoxy-3-(2-methylpropyl)-1,3,4,6,7,11b-hexahydrobenzo [a]quinolizin-2-one, Cat# 2175) were dissolved in dimethyl sulfoxide (DMSO) and were obtained from Tocris Bioscience (Minneapolis, MN, United States). The reversible VMAT-2 inhibitor VBZ (IUPAC name: (2R,3R,11bR)-3-Isobutyl-9,10-dimethoxy-1,3,4,6,7,11b-hexahydro-2H-pyrido[2,1-a]isoquinolin-2-yl L-valinate, Cat# HY-16771) was purchased from MedChemExpress (Monmouth Junction, NJ, United States). Sodium channel blocker tetrodotoxin citrate (IUPAC name: (1R,5R,6R,7R,9S,11S,12S,13S,14S)-3-amino-14-(hydroxymethyl)-8,10-dioxa-2,4 diazatetracyclo[7.3.1.17,11.01,6]tetradec-3-ene-5,9,12,13,14-pentol, Cat# 1069) was obtained from Alomone labs Ltd. (Jerusalem, Israel). Trichloroacetic acid (Cat# T0669) was purchased from Merck KGaA (Darmstadt, Germany). Isoflurane (Cat# 1214) was obtained from Medicus Partner Ltd. (Biatorbágy, Hungary). Other materials used for experiments were purchased from general commercial resources and were of the highest grade.

### 2.3 Resting and axonal stimulation-evoked release of [^3^H] NA and [^3^H] 5-HT from *ex vivo* hippocampal slice preparation

**Figure 1:**
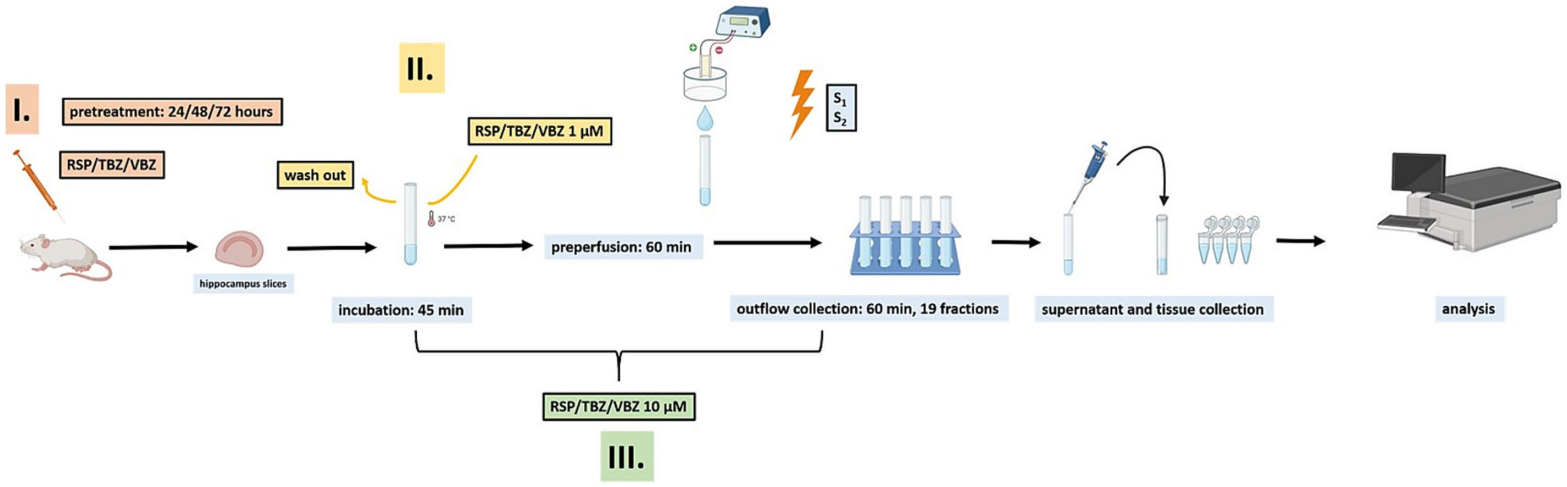
Schematic representation of the experimental workflow. Hippocampal slices were incubated with [^3^H] noradrenaline ([^3^H] NA) or [^3^H] serotonin ([^3^H] 5-HT) in Krebs solution, followed by preperfusion to remove extracellular radioactivity. Outflow fractions were then collected during resting conditions and electric field stimulation (S_1_, S_2_), and after the experiment both supernatant and tissue were collected for analysis. Drugs [reserpine (RSP), tetrabenazine (TBZ), and valbenazine (VBZ)] were applied in three different ways: (i) intraperitoneal pretreatment 24–72 h before slice preparation, (ii) added only during a 45-min incubation phase and then washed out, or (iii) continuously present in the perfusate until the end of the measurement.

**Figure 2:**
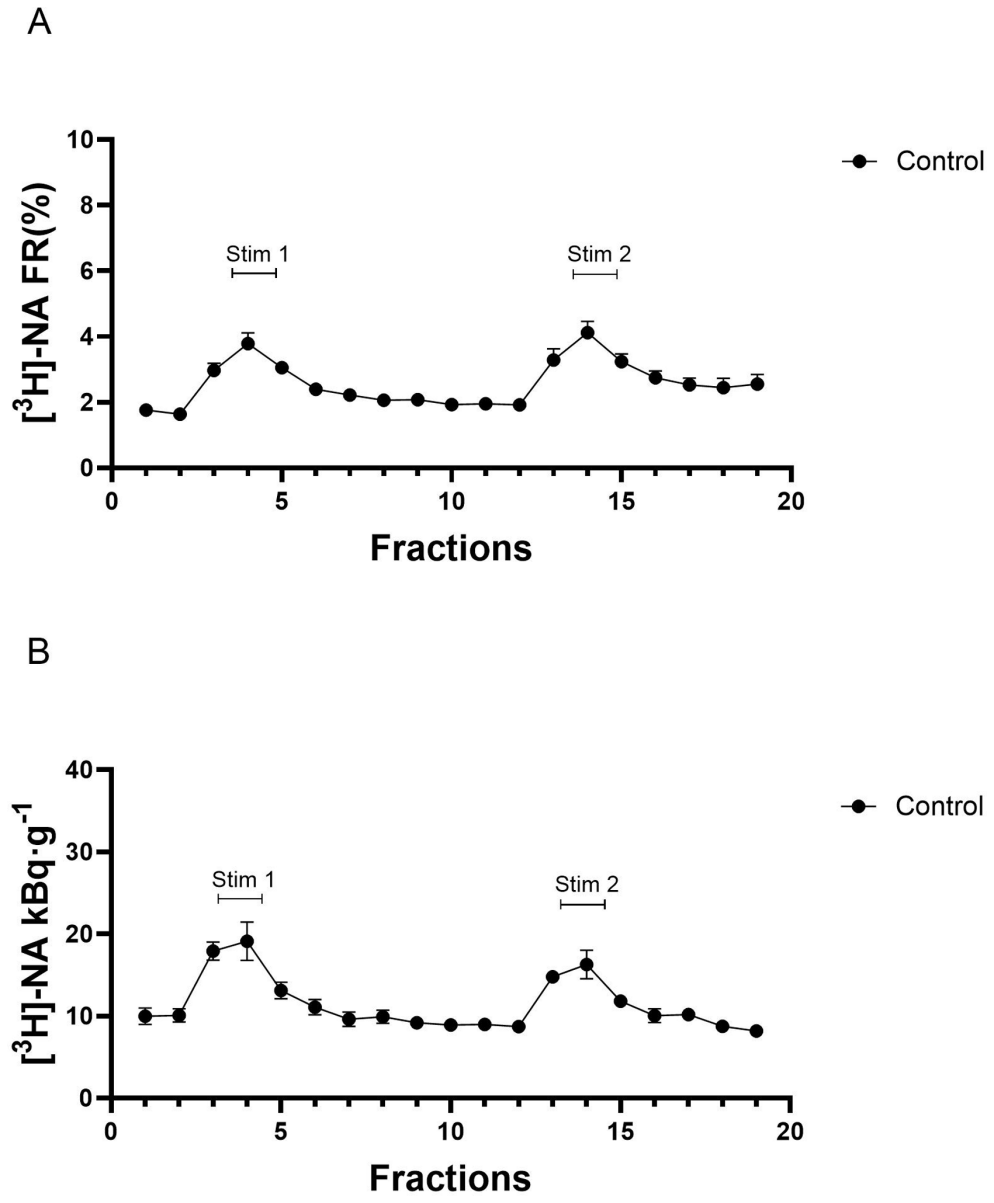
The release of [^3^H] noradrenaline ([^3^H] NA) in response to electric field stimulations expressed in fractional release **(A)** and Bq·g^−1^ **(B)**. The tissue slices were stimulated at the third (Stim 1) and 13th (Stim 2) fractions. Curves show the means ± SEM of the identical experiments. *n* = 6 mice.

**Figure 3:**
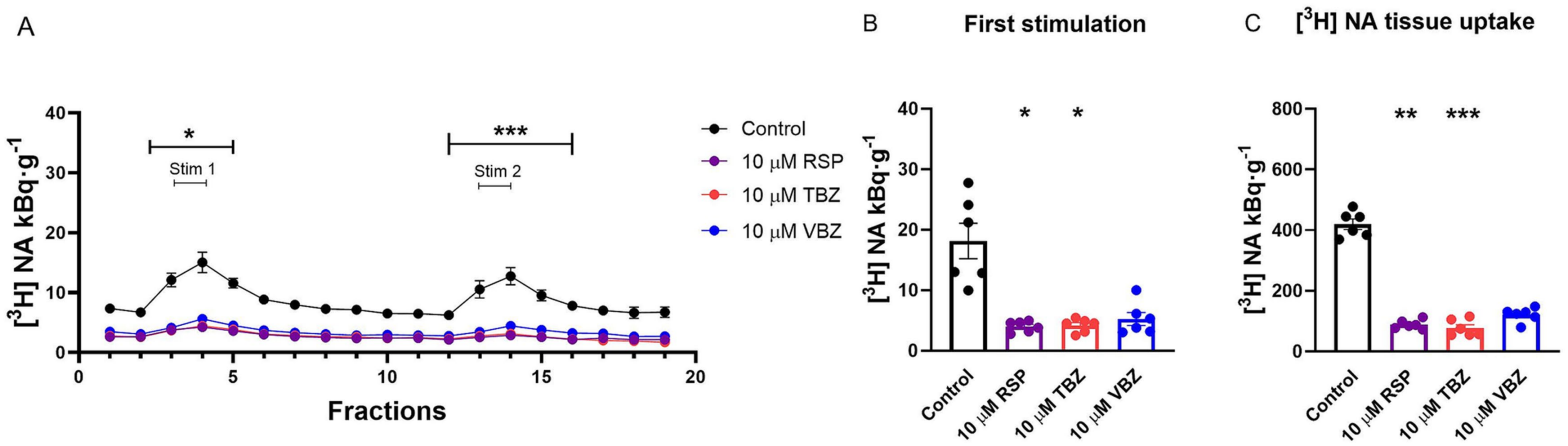
Effect of reserpine (RSP), tetrabenazine (TBZ), and valbenazine (VBZ) on [^3^H] noradrenaline ([^3^H] NA) release and uptake in the mouse hippocampus. Tissue slices were incubated in Krebs solution; RSP, TBZ, and VBZ (10 μM) were introduced during the incubation and were allowed to remain until the end of the experiment. The tissue slices were stimulated at the third (Stim 1) and 13th (Stim 2) fractions. The released radioactivity was measured in the collected fractions. **(A)** [^3^H] NA release in response to electrical stimulation (S_1_ and S_2_). The efflux of [^3^H] NA was decreased in the presence of RSP and TBZ throughout the experimental period, and also by VBZ regarding the second stimulus, compared with the control group. **(B)** [^3^H] NA release in response to the first electrical stimulation (S_1_). The application of 10 μM RSP and TBZ markedly decreased the [^3^H] NA release upon electric field stimulation. **(C)** [^3^H] NA uptake into tissue. 10 μM RSP and TBZ significantly reduced [^3^H] NA uptake into tissue slices. Curves show the means ± SEM of the identical experiments. *n* = 6 mice/group. **(A,B)** Kruskal-Wallis test followed by Dunn’s multiple comparisons *post hoc* test (**(A,B)** S_1_: *F* [3, 24] = 12.95, *p* < 0.05) or **(A)** One-way ANOVA with square root transformation followed by Tukey’s multiple comparisons *post hoc* test (S_2_: *F* [3, 20] = 18.12, *p* < 0.05) **(C)** Kruskal-Wallis test followed by Dunn’s multiple comparisons *post hoc* test (*F* [3, 24] = 17.45, *p* < 0.05). *p < 0.05 compared to the control group. [^3^H], tritium; NA, noradrenaline; RSP, reserpine; TBZ, tetrabenazine; VBZ, valbenazine; S_1_, first electric field stimulation; S_2_, second electric field stimulation.

**Figure 4:**
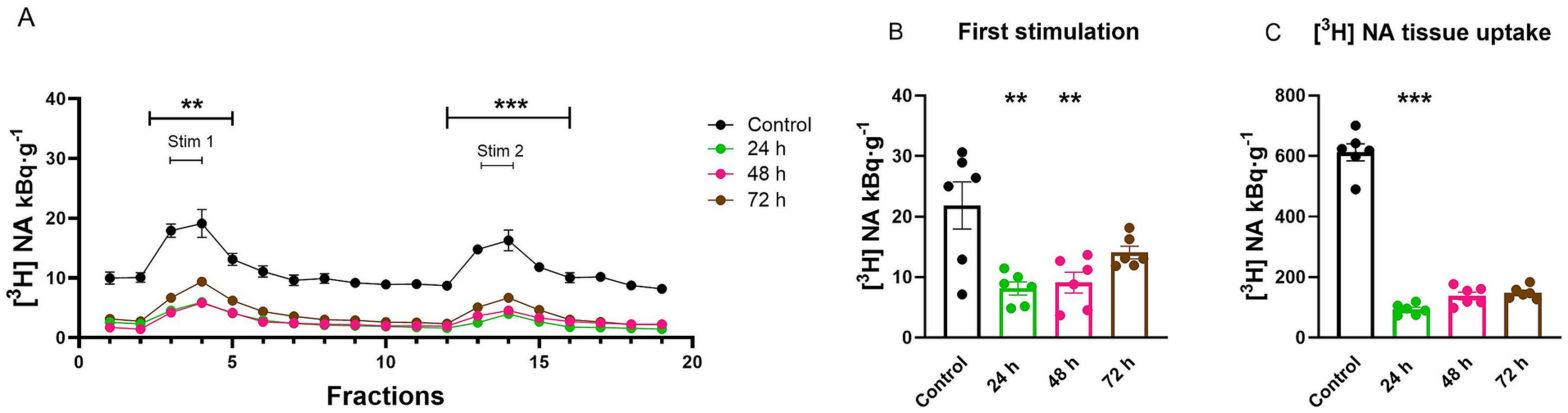
The persistent long-lasting effect of reserpine (RSP, 1 mg kg^−1^, i.p.) on the resting and electric field stimulated-evoked release of [^3^H]-noradrenaline ([^3^H] NA) in *ex vivo* hippocampal slices. Control and after 24, 48, and 72 h after treatment. Tissue slices were incubated in Krebs solution; mice were treated with RSP i.p. 24, 48, or 72 h before the experiment. The tissue slices were stimulated at the third (Stim 1) and 13th (Stim 2) fractions. The released radioactivity was measured in the collected fractions. **(A)** [^3^H] NA release in response to electrical stimulation (S_1_ and S_2_). The efflux of [^3^H] NA was decreased in the presence of RSP treatment after 24 and 48 h during the entire experimental period, but after 72 h, there was no significant decrease in the [^3^H] NA efflux after S_1_, only after S_2_, compared with the control group. **(B)** [^3^H] NA release in response to the first electrical stimulation (S_1_). The administration of RSP markedly decreased the [^3^H] NA release upon electric field stimulation 24 and 48 h after treatment, but this effect was not seen after 72 h. **(C)** [^3^H] NA uptake into tissue. Only 24 h after RSP treatment did we see a reduction in [^3^H] NA uptake into tissue slices, compared with the control group. Curves show the means ± SEM of the identical experiments. *n* = 6 mice/group. **(A,B)** One-way ANOVA with square root transformation followed by Tukey’s multiple comparisons *post hoc* test (**(A,B)** S_1_: *F* [3, 20] = 7.71, *p* < 0.05, **(A)** S_2_: *F* [3, 20] = 14.93, *p* < 0.05) and **(C)** Kruskal-Wallis test followed by Dunn’s multiple comparisons *post hoc* test (*F* [3, 24] = 18.6, *p* < 0.05). *p < 0.05 compared to the control group. [^3^H], tritium; NA, noradrenaline; RSP, reserpine; S_1_, first electric field stimulation; S_2_, second electric field stimulation.

**Figure 5:**
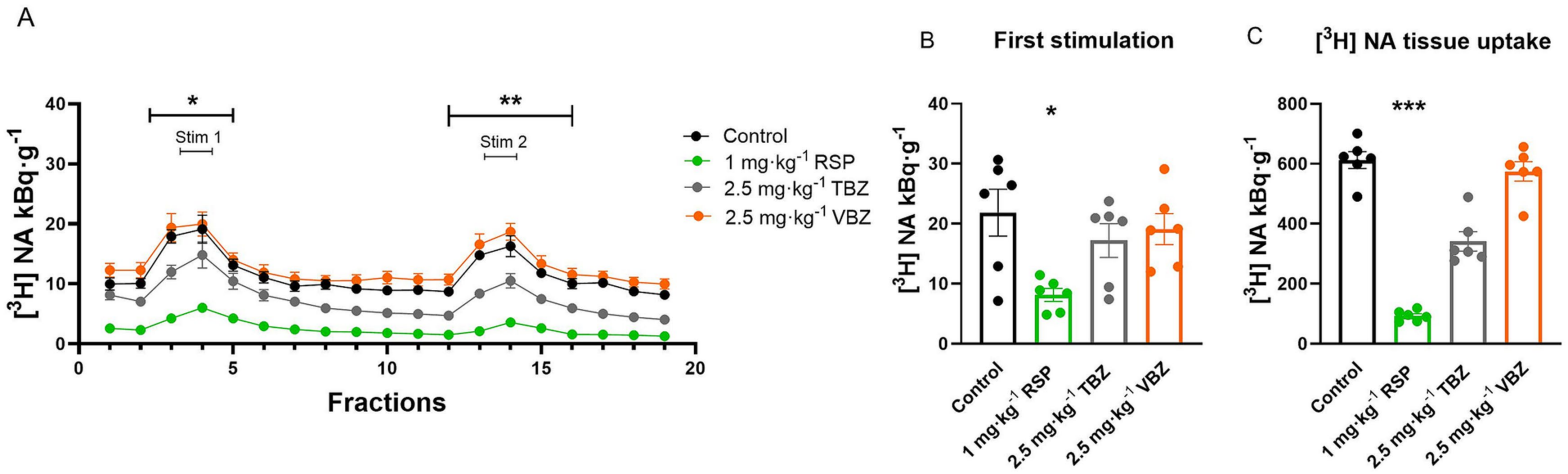
Effect of reserpine (RSP, 1 mg kg^−1^), tetrabenazine (TBZ, 2.5 mg kg^−1^), and valbenazine (VBZ, 2.5 mg kg^−1^) pretreatment on [^3^H] noradrenaline ([^3^H] NA) release and uptake in the mouse hippocampus. Tissue slices were incubated in Krebs solution and stimulated at the third (Stim 1) and 13th (Stim 2) fractions. The released radioactivity was measured in the collected fractions. **(A)** [^3^H] NA release in response to electrical stimulation (S_1_ and S_2_). The efflux of [^3^H] NA was decreased after RSP treatment throughout the experiment upon electric field stimulation, while TBZ and VBZ did not influence it. **(B)** [^3^H] NA release in response to the first electrical stimulation (S_1_). The administration of RSP inhibited the [^3^H] NA release upon electric field stimulation, while TBZ and VBZ treatment had no significant effect. **(C)** [^3^H] NA uptake into tissue slices. RSP treatment significantly reduced the tissue’s [^3^H] NA uptake, in contrast to TBZ and VBZ, which did not alter it. Curves show the means ± SEM of the identical experiments. *n* = 6 mice/group. **(A,B)** One-way ANOVA followed by Tukey’s multiple comparisons *post hoc* test (**(A,B)** S_1_: *F* [3, 20] = 4.54, *p* < 0.05; S_2_: *F* [3, 20] = 7.95, *p* < 0.05); or **(C)** Kruskal-Wallis test followed by Dunn’s multiple comparisons *post hoc* test (*F* [3, 24] = 19.46, *p* < 0.05). *p < 0.05 compared to the control group. [^3^H], tritium; NA, noradrenaline; RSP, reserpine; TBZ, tetrabenazine; VBZ, valbenazine; S_1_, first electric field stimulation; S_2_, second electric field stimulation.

**Figure 6:**
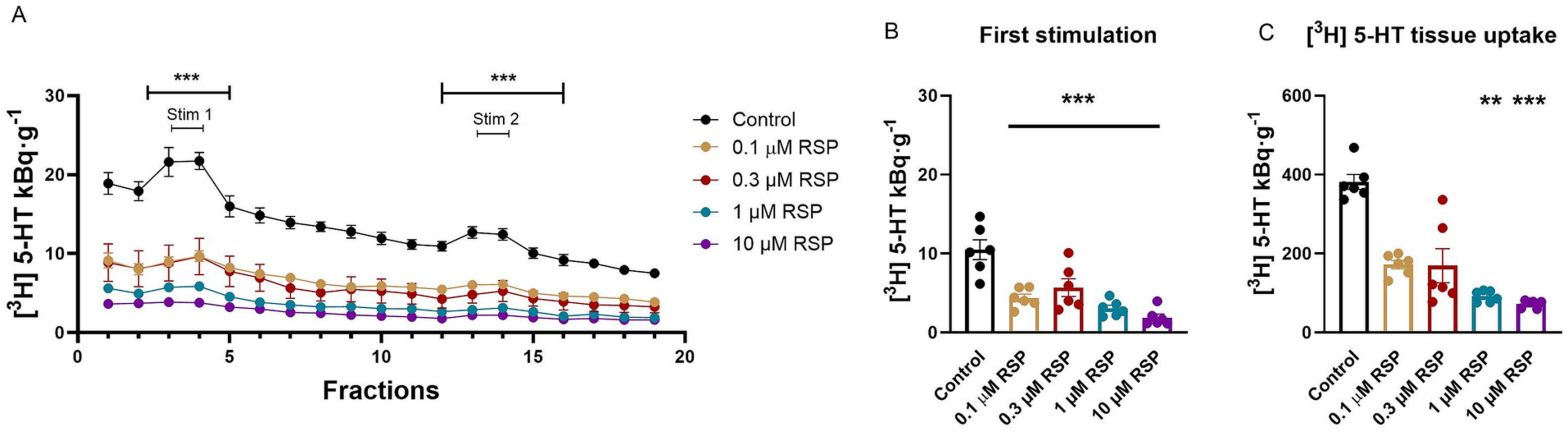
Effect of reserpine (RSP) on [^3^H] serotonin ([^3^H] 5-HT) release and tissue uptake in the mouse hippocampus. Tissue slices were incubated in Krebs solution; RSP (0.1, 0.3, 1.0, and 10 μM) was introduced during the incubation and remained until the end of the experiment. The tissue slices were stimulated at the third (Stim 1) and 13th (Stim 2) fractions. The released radioactivity was measured in the collected fractions. **(A)** [^3^H] 5-HT release in response to electrical stimulation (S_1_ and S_2_). The efflux of [^3^H] 5-HT was decreased in the presence of all applied doses of RSP. **(B)** [^3^H] 5-HT release in response to the first electrical stimulation (S_1_). The administration of RSP inhibited the [^3^H] 5-HT release upon electric field stimulation at all applied doses significantly. **(C)** [^3^H] 5-HT uptake into tissue slices. One and ten micrometer RSP reduced [^3^H] 5-HT uptake into tissue slices. Other concentrations did not change it significantly. Curves show the means ± SEM of the identical experiments. *n* = 6 mice/group. **(A,B)** One-way ANOVA with square root transformation followed by Tukey’s multiple comparisons *post hoc* test (**(A,B)** S_1_: *F* [4, 25] = 16.04, *p* < 0.05; **(A)** S_2_: *F* [4, 25] = 14.99, *p* < 0.05) and **(C)** Kruskal-Wallis test followed by Dunn’s multiple comparisons *post hoc* test (*F* [4, 30] = 23.73, *p* < 0.05). *p < 0.05 compared to the control group. [^3^H], tritium; 5-HT, serotonin; RSP, reserpine; S_1_, first electric field-stimulation; S_2_, second electric field-stimulation.

**Figure 7:**
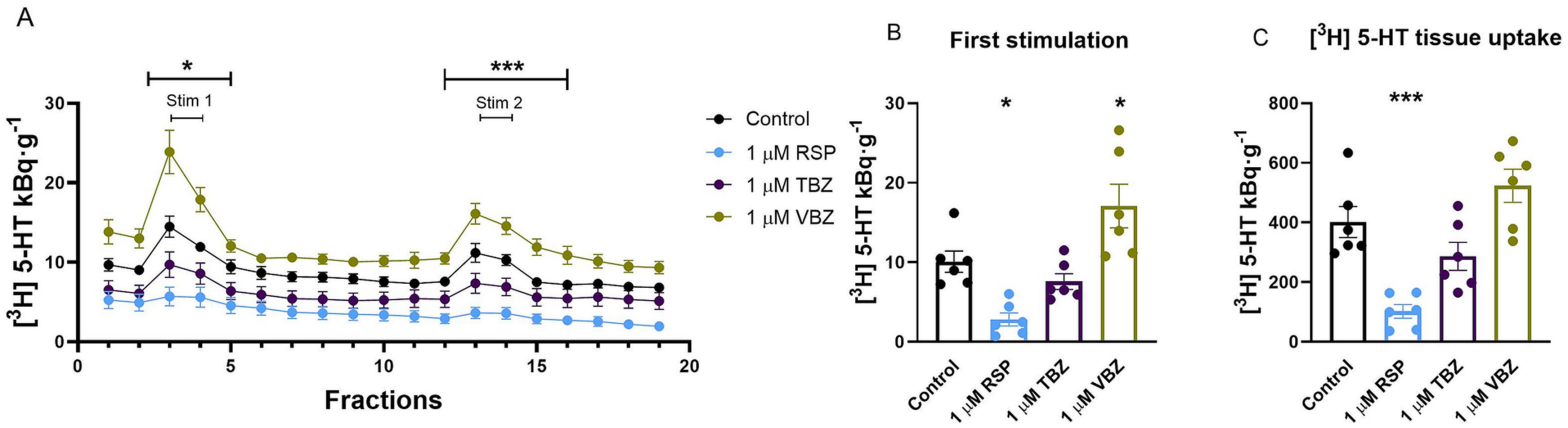
Effect of reserpine (RSP), tetrabenazine (TBZ), and valbenazine (VBZ) on [^3^H] serotonin ([^3^H] 5-HT) release and tissue uptake in the mouse hippocampus. Tissue slices were incubated in Krebs solution; RSP, TBZ, and VBZ (1 μM) were introduced only during the incubation and were not allowed to remain until the end of the experiment. The tissue slices were stimulated at the third (Stim 1) and 13th (Stim 2) fractions. The effect of RSP was washout resistant. The released radioactivity was measured in the collected fractions. **(A)** [^3^H] 5-HT release in response to electrical stimulation (S_1_ and S_2_). During the incubation phase, the presence of RSP decreased the efflux of [^3^H] 5-HT, while in contrast, the availability of VBZ increased the [^3^H] 5-HT release during the entire experiment. The presence of TBZ did not significantly alter [^3^H] 5-HT release upon S_1_ and S_2_. **(B)** [^3^H] 5-HT release in response to the first electrical stimulation (S_1_). The administration of RSP during incubation phase markedly decreased the [^3^H] 5-HT release upon the first electric field stimulation. However, adding VBZ increased the [^3^H] 5-HT efflux in response to S_1_. **(C)** [^3^H] 5-HT uptake into tissue slices. One micrometer RSP reduced [^3^H] 5-HT uptake into tissue slices, in contrast to TBZ and VBZ, which did not change it. Curves show the means ± SEM of the identical experiments. *n* = 6 mice/group. **(A–C)** One-way ANOVA with square root transformation **(A,B)** followed by Tukey’s multiple comparisons *post hoc* test (**(A,B)** S_1_: *F* [3, 20] = 12.94, *p* < 0.05; S_2_: *F* [3, 20] = 18.46, *p* < 0.05; **(C)** *F* [3, 20] = 15.07, *p* < 0.05). *p < 0.05 compared to the control group. [^3^H], tritium; 5-HT, serotonin; RSP, reserpine; TBZ, tetrabenazine; VBZ, valbenazine; S_1_, first electric field-stimulation; S_2_, second electric field-stimulation.

**Figure 8:**
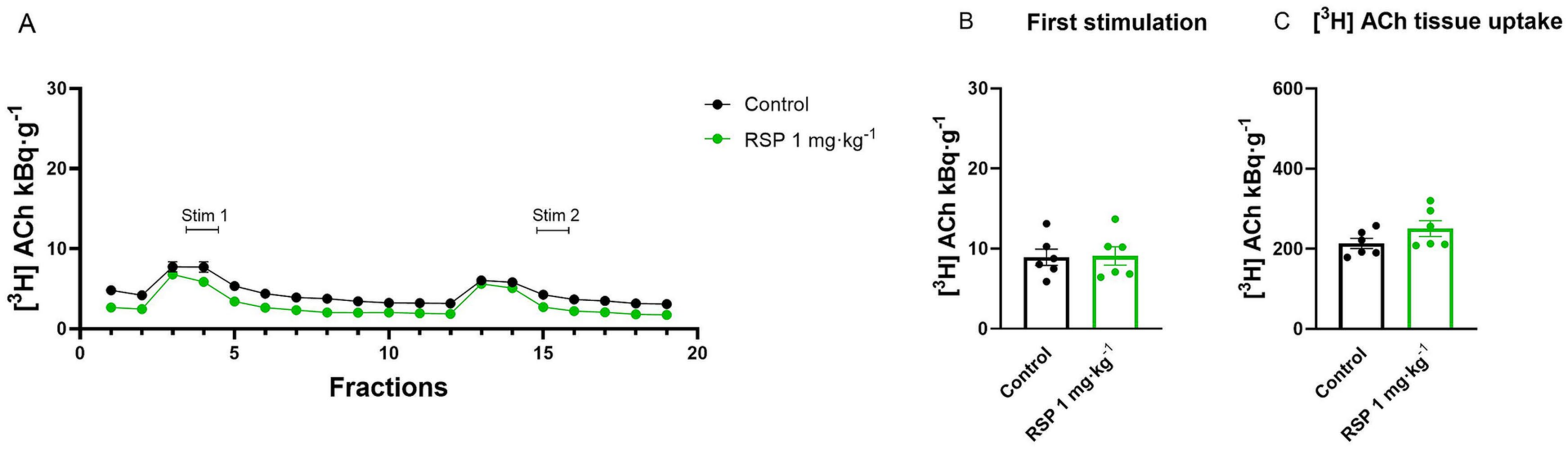
Reserpine (RSP) fails to influence the release of [^3^H] ACh from hippocampal slice preparations. Tissue slices were incubated in Krebs solution; mice were treated with 1 mg/kg RSP 24 h before the experiment. The tissue slices were stimulated at the third (Stim 1) and 13th (Stim 2) fractions. The released radioactivity was measured in the collected fractions. **(A)** [^3^H] ACh release in response to electrical stimulation (S_1_ and S_2_). There is no significant difference in pretreatment over time. **(C)** [^3^H] ACh uptake into tissue. 1 mg·kg^−1^ RSP pretreatment did not alter [^3^H] ACh uptake into tissue slices. Curves show the means ± SEM of the identical experiments. *n* = 6 mice/group. **(A–C)** Student’s *t*-test [**(A,B)** S_1_: *t* = 0.1034, df = 10, *p* = 0.8005; **(B)** S_2_: *t* = 1.2144, df = 10, *p* = 0.7499; **(C)** *t* = 1.576, df = 10, *p* = 0.3723]. [^3^H], tritium; ACh, acetylcholine; RSP, reserpine; S_1_, first electric field stimulation; S_2_, second electric field stimulation.

Mice were sacrificed in a 2-step manner according to the American Veterinary Medical Association guidelines for the Euthanasia of Animals. First, the animals were anaesthetized with a relatively high dose of isoflurane for rapid loss of consciousness (>1.5 v/v %) in a sufficiently large container to prevent hypoxia. Then, mice were decapitated, and the brain was immediately removed and placed into ice-cold Krebs solution [composition (in mM): 113 NaCl, 4.7 KCl, 1.2 MgSO_4_, 2.5 CaCl_2_, 25 NaHCO_3_, 1.2 KH_2_PO_4_, 115 glucose, 0.3 Na_2_EDTA, and 0.03 ascorbic acid]. After removal, the hippocampus was isolated and sliced into 400 μm thick slices (Milusheva et al., 1994) using a tissue chopper and incubated for 45 min in 1 mL of Krebs buffer (see above) containing radioactive [^3^H] NA or [^3^H] 5-HT (5 μCi·mmol^−1^). After this incubation period, the incubation medium was replaced, allowing complete washout of any residual volatile anesthetic. Although we did not directly measure the residual isoflurane concentration, similar incubation and washout protocols have been applied in our previous studies (Csölle et al., 2013; Tod et al., 2024; Gölöncsér et al., 2024), where animals were anesthetized using the same procedure and no confounding effects on neurotransmitter uptake or release were observed. Therefore, it is unlikely that isoflurane exposure affected the present results. Possible residual effects of isoflurane anesthesia were controlled by the incubation and washout procedures described in the Discussion (see Discussion, Limitations, Methodological considerations). Experiments were conducted at 37 °C in a modified Krebs solution, continuously saturated with carbogen gas (95% O_2_ and 5% CO_2_). To investigate the effects of RSP, TBZ, and VBZ, three different treatment protocols were applied (Figure 1). (i) In some experiments, mice received intraperitoneal injections of the corresponding drug (RSP 1 mg·kg^−1^, TBZ 2.5 mg·kg^−1^, VBZ 2.5 mg·kg^−1^) 24, 48, or 72 h before hippocampal slices were prepared for release measurements (Figures 4, 5, 8). (ii) In other experiments, the drugs were added only during a 45-min incubation period in 1 mL Krebs solution, after which they were washed out and were no longer present during the measurement (Figure 7). (iii) In a third set of experiments, the drugs were continuously present in the Krebs perfusate until the end of the measurement (Figures 2, 3, 6). Following the incubation, the preparations were washed three times with 10 mL of ice-cold, oxygenated Krebs solution, and three to four slices were transferred to a thermoregulated four-channel (internal volume, 100 μL) microperfusion system (Hennings et al., 1999) and maintained at 37 °C. The release experiments were performed as previously described (Milusheva et al., 2008); the preparation was superfused with Krebs solution at a rate of 0.5 mL·min^−1^ for 60 min before each measurement. After 60 min of preperfusion, the outflow was collected in 1.5 mL·min^−1^ fractions (3 min each) for 60 min (19 fractions). During the third (S_1_) and 13^th^ (S_2_) collection periods, the preparations were field-stimulated electrically with the following parameters: [^3^H] NA: 20 V, 2 Hz, with a 2 ms impulse duration for 1 min (120 pulses) and [^3^H] 5-HT: 40 V, 2 Hz, with a 2 ms impulse duration for 1 min (120 pulses) using a Grass S88 stimulator. The vesicular release of radioactivity evoked by electric field stimulation was sensitive to tetrodotoxin, which was applied at a concentration of 1 μM. The supernatant (500 μL) from each fraction was added to 2 mL of scintillation mixture (Ultima Gold; Packard, Canberra, Australia). At the end of the perfusion period, the tissue was removed from the chamber and the residual radioactively labeled neurotransmitters were extracted with 5 mL of 10% trichloroacetic acid for 30 min; 100 μL of the supernatant was added to 2 mL of scintillation mixture, and the radioactivity was measured using a Packard 1900 Tricarb and 5110 TR liquid scintillation counter (Packard). The samples’ radioactivity was expressed in disintegrations per gram of wet tissue weight (Becquerels per gram, Bq·g^−1^).

The tissue’s [^3^H] NA and [^3^H] 5-HT uptake by the tissue slices was quantified as the amount of radioactivity present in the tissue at the beginning of the perfusion period (CB), estimated using Equation (1):


∑i1–19FRi+CE=CB (1)

where FRi corresponds to the radioactivity detected in the i-th fraction, and CE refers to the remaining radioactivity in the tissue measured at the end of the experiment.

The release evoked by electrical field stimulation (S_1_ and S_2_) was calculated as the total radioactivity released above the baseline (resting) levels, represented by R1 and R2, respectively. R1 was defined as the average radioactivity measured in the first and second fractions, while R2 corresponded to the average radioactivity in the 14th and 15th fractions. A custom-made equation was used, and the radioactivity measured in the sample was calculated as the fractional release (FR) Equation 2:


$$\mathrm{FR}\%=\frac{\text{released tritium in}\;\mathrm{Bq}/g\times 100}{\begin{array}{l} \text{tritium in}\;\mathrm{Bq}/g\;\text{in the tissue}\;\mathrm{at}\;\text{the}\;\\ \;\;\;\;\;\;\text{time of sample measurement} \end{array}}$$ (2)

The release in response to electric field stimulation was evaluated in fractional release (FR) and as the ratio of the area under the curve of the total radioactivity release to the resting in response to supramaximal electrical field stimulation, applied during the third (S_1_) and 13^th^ (S_2_) fractions, in the absence (FRS1) and presence (FRS2) of the drug. The FRS2/FRS1 ratio was then calculated to evaluate the drug’s effect, with the S1-evoked release serving as the internal standard. Similarly, the impact on resting release was assessed using the FRR2/FRR1 ratio, where FRR1, defined as the average radioactivity from the first and second fractions, served as the internal reference, unless stated otherwise. Previous studies reported that 88% of the stimulation-induced increase in [^3^H] release under identical experimental conditions was due to the increased release of [^3^H] NA (Vizi and Burnstock, 1988), as well as [^3^H] 5-HT.

### 2.4 Release of [^3^H] ACh from *ex vivo* hippocampal slices

There is several evidence that the cholinergic system is also involved in the pathophysiology of depression through nAChR alpha7 activation (Pahlavani, 2024); therefore, we investigated the effect of RSP on the stimulation-evoked release of acetylcholine. For the detection of [^3^H] ACh release, hippocampal slices were obtained as described for [^3^H] NA. First, we conducted a 45-min-long incubation with [^3^H]-choline (5 μCi/mL) in a 1 mL organ bath containing 37 °C Krebs solution. At the end of the incubation period, the slices were washed five times with Krebs solution and transferred to the four-channel microperfusion system and superfused with Krebs solution at a rate of 0.5 mL/min for 60 min. To selectively prevent the [^3^H] choline reuptake, high-affinity choline transporter inhibitor hemicholinium-3 (10 μM) was added throughout the experiments, which did not affect ACh’s resting and axonal stimulation-induced release. After 60 min of preperfusion, nineteen 3-min samples were collected, and the tissues were stimulated twice, at the third fraction (S_1_) and the 13th fraction (S_2_). Supramaximal electrical train stimulation was performed using a Grass S88 stimulator, with the following parameters: 40 V, 2 Hz, 2 ms, 1 min. The total radioactivity released from the tissue and collected in the perfusion fluid was considered to represent the amount of vesicular [^3^H] ACh released following hydrolysis rather than that of [^3^H] choline released directly from nerve terminals (Milusheva et al., 1994). At the end of radioactivity collection, supernatant (500 μL) from each fraction was added to 2 mL of scintillation mixture (Ultima Gold; Packard). After sample collection, the tissues were removed from the chambers, and the residual [^3^H] ACh was extracted using 5 mL of 10% trichloroacetic acid for 30 min. Subsequently, 100 μL of supernatant was added to 2 mL of scintillation mixture; the radioactivity was determined using a liquid scintillation counter (Packard 1900 Tricarb and 5110 TR) and expressed in disintegrations per gram of wet tissue weight (Bq·g^−1^).

The release of [^3^H] ACh at rest and in response to axonal stimulation was calculated in FR and Bq·g^−1^ as described for NA and 5-HT. The effect of axonal stimulation or drugs on the release was evaluated as the ratio of the area under the curve of the total radioactivity release to the resting release in response to the first and second stimulation (FRS2/FRS1 or S_2_/S_1_ in Bq·g^−1^). A similar calculation was performed for the resting release (FRR2/FRR1 or S_2_/S_1_ in Bq·g^−1^).

### 2.5 Statistical analysis

We calculated the animal numbers required for group sizes using G*Power 3.1.9.7 software, as shown above. Values in the paper were expressed as the mean ± SEM (error bars). Six animals were used in each experimental group. The normality of the experimental data distribution was tested using the Shapiro–Wilk normality test. Depending on the datasets, statistical analyses were performed with unpaired Student’s *t*-test or one-way ANOVA with Tukey’s *post hoc* test if the data were normally distributed; if not, Kruskal-Wallis test with multiple comparisons with Dunn’s multiple comparisons *post hoc* test using the STATISTICA version 14.0.1 software (TIBCO Software Inc., Palo Alto, CA, United States). *Post hoc* tests were only performed when F in ANOVA achieved *p* < 0.05. *p*-values of less than 0.05 were considered statistically significant throughout the study.

## 3 Results

### 3.1 Effects of reserpine, tetrabenazine, and valbenazine on resting and electric field stimulation-evoked vesicular release of radioactivity from hippocampus slice preparations loaded with [^3^H] NA

After loading the tissue with [^3^H] NA and washout, we measured the resting and electric field stimulation-evoked vesicular release of [^3^H] NA from hippocampus slice preparations. Figure 2 shows the release of [^3^H] NA during resting and in response to electric stimulation. In Figure 2A, the radioactivity content of each perfusate sample was expressed as a percentage of the tissue content calculated at the time of sampling (FR%). Figure 2B represents the release calculated in Bq·g^−1^. The resting release in the first 3-min collection period was 12.66 ± 2.63 kBq·g^−1^, and it was relatively constant from one collection period to the next throughout the experiments. Radioactivity released by the first stimulation (S_1_) was 18.20 ± 2.94 kBq·g^−1^. The release of radioactivity in two consecutive stimulation periods (FRS2/FRS1) was 0.98 ± 0.04 in control experiments (Figure 2A), indicating that the amount of fractional release in response to consecutive electric field stimulations remained relatively constant. The tissue’s [^3^H] NA content was 420.0 ± 17.1 kBq·g^−1^. The release during resting was also constant: FRR2 ± FRR1 = 0.93 ± 0.03. In the presence of tetrodotoxin (1 μM), there was no significant increase in [^3^H] NA release in response to electric stimulation, indicating that the evoked release of tritium is of neuronal origin and depends on the propagation of action potentials along the neuronal membrane. This is a well-established basic finding that has been demonstrated previously by others (Raiteri et al., 1990; Westphalen et al., 2009) and also in our earlier work (Török et al., 2004; Tod et al., 2024). The effects of RSP, a competitive inhibitor of the VMAT-2 enzyme (Wu et al., 2024), were measured on electric field stimulation-evoked vesicular release (Figure 3A) and storage capacity (Figure 3B) of *ex vivo* hippocampal slices. To demonstrate that VMAT-2 is responsible for transporting transmitters into vesicles at axon terminals and that its inhibition results in a reduction of quantal, exocytotic transmitter release evoked by axonal stimulation, the effects of TBZ and VBZ, non-competitive selective VMAT-2 inhibitors (Eiden et al., 2004) were also studied and compared to RSP loaded and control groups in these preparations (Figure 3).

RSP and the other two antagonists were administered at a 10 μM concentration during the tissue loading and kept in the solution. RSP and TBZ significantly reduced the electric field stimulation-evoked [^3^H] NA vesicular release throughout the experiment (RSP: S_1_: 4.0 ± 0.4 kBq·g^−1^; *p* = 0.0115; S_2_: 1.1 ± 0.3 kBq·g^−1^; *p* < 0.0001; TBZ: S_1_: 4.2 ± 0.5 kBq·g^−1^; *p* = 0.0132; S_2_: 2.1 ± 0.6 kBq·g^−1^; *p* = 0.0001). The presence of VBZ decreased the release significantly, exclusively following the second stimulation (S_1_: 5.3 ± 1.1 kBq·g^−1^; *p* = 0.0766; S_2_: 2.8 ± 1.2 kBq·g^−1^; *p* < 0.0001), compared to the control group (S_1_: 18.2 ± 2.9 kBq·g^−1^; S_2_: 13.7 ± 2.4 kBq·g^−1^) (Figures 3A,B). As far as the storage capacity is concerned, 10 μM RSP (88.9 ± 6.0 kBq·g^−^1: *p* = 0.0065) and TBZ (77.1 ± 11.2 kBq·g^−1^: *p* = 0.0009) significantly reduced the tissue’s [^3^H] NA content, compared to the control group (420.0 ± 17.2 kBq·g^−1^) (Figure 3C). VBZ (121.0 + 9.4 kBq·g^−1^) did not alter the tissue’s [^3^H] NA uptake. Compared to the RSP-loaded group, there was no significant difference neither at the electric field stimulation-evoked [^3^H] NA vesicular release nor at the tissue’s [^3^H] NA uptake regarding the TBZ and VBZ loaded groups.

### 3.2 Persistent effects of reserpine on vesicular [^3^H] NA release

To explore the length of the inhibitory effect of RSP (1 mg·kg^−1^, i.p.) on the vesicular release of [^3^H] NA and storage capacity in hippocampal slices, we studied its action (Figure 4), 24, 48, and 72 h after RSP i.p. treatment. The slices were loaded with [^3^H] NA and maintained throughout the experiments.

RSP administrations’ effect on vesicular release and storage capacity of [^3^H] NA proved to be irreversible (Figure 4). The administration of RSP inhibited the electric field stimulation-evoked release of [^3^H] NA 24, and even after 48 h after treatment (24 h: S_1_: 8.1 ± 10.7 kBq·g^−1^; *p* = 0.018; S_2_: 4.2 ± 0.7 kBq·g^−1^; *p* < 0.0001; 48 h: S_1_: 9.1 ± 1.7 kBq·g^−1^; *p* = 0.0037; S_2_: 5.2 ± 0.6 kBq·g^−1^; *p* = 0.0001) compared to the control group (S_1_: 21.8 ± 3.4 kBq·g^−1^; S_2_: 15.5 ± 2.4 kBq·g^−1^). However, after 72 h, a decreased [^3^H] NA efflux was seen exclusively after the S_2_, while after S_1_ there was no difference (S_1_: 14.0 ± 1.0 kBq·g^−1^; *p* = 0.1020; S_2_: 9.3 ± 0.5 kBq·g^−1^; *p* = 0.0139), compared to the control group (Figures 4A,B). Twenty-four hours after RSP treatment, the tissue’s [^3^H] NA content was reduced significantly (93.0 ± 7.2 kBq·g^−1^; *p* = 0.0001), compared to the control group (612.0 ± 28.3 kBq·g^−1^), indicating that 84.9% of NA is not stored in vesicles. Forty-eight hours and seventy-two hours after RSP administration, the radioactivity measured in the tissue (138 ± 12.6and 148 ± 8.4 kBq·g^−1^; *p* = 0.0766 and *p* = 0.2474) was not significantly reduced compared to the control group (Figure 4C).

### 3.3 Effects of tetrabenazine, valbenazine, and reserpine i.p. pretreatment on resting and electric field stimulation-evoked vesicular release of [^3^H] NA

We studied the effects of TBZ, VBZ, and RSP treatments 24 h after i.p. administration. We measured the axonal stimulation-evoked release of radioactivity from slice preparations loaded with [^3^H] NA in response to RSP (1 mg·kg^−1^), TBZ (2.5 mg·kg^−1^), and VBZ (2.5 mg·kg^−1^) administration, expressed in Bq·g^−1^ (Figure 5A). The pre-administration of RSP inhibited the S_1_ and S_2_ induced release of [^3^H] NA from the hippocampus (S_1_: 8.1 ± 10.7 kBq·g^−1^; *p* = 0.0116; S_2_: 4.2 ± 0.7 kBq·g^−1^; *p* = 0.0029) compared to the control group (S_1_: 21.8 ± 3.4 kBq·g^−1^; S_2_: 15.5 ± 2.4 kBq·g^−1^), indicating that its effect is long-lasting and irreversible. The pre-administration of TBZ (S_1_: 17.2 ± 2.8 kBq· g^−1^; *p* = 0.6463 and S_2_: 12.7 ± 2.1 kBq·g^−1^; *p* = 0.7389) and VBZ (S_1_: 19.1 ± 2.6 kBq·g^−1^; *p* = 0.8964; S_2_: 16.1 ± 2.2 kBq·g^−1^; *p* = 0.9953) failed to reduce the electric field stimulation-evoked release of [^3^H] NA, compared to the non-pretreated control group (Figures 5A,B). RSP pretreatment significantly reduced the tissue’s [^3^H] NA content (93.0 ± 7.2 kBq·g^−1^; *p* = 0.0004), while the administration of VBZ and TBZ did not alter it (341.0 ± 32.5 and 574.0 ± 32.4 kBq·g^−1^; *p* = 0.0858 and *p* > 0.9999), compared to the control group (612.0 ± 28.3 kBq·g^−1^) (Figure 5C). The administration of the RSP dose was very efficient and reduced both vesicular release of [^3^H] NA and the tissue’s [^3^H] NA uptake by 62.9 and 84.9%, respectively. TBZ and VBZ had no reducing effect (Figure 5), indicating that their action was short-lasting and reversible.

### 3.4 Effects of reserpine on electric field stimulation-evoked vesicular release of [^3^H] 5-HT from *ex vivo* hippocampal slices

In further experiments, we studied the stimulation-evoked vesicular release of radioactivity from slices loaded with [^3^H] 5-HT in the presence of 0.1, 0.3, 1.0, and 10 μM RSP (Figure 6). All applied doses significantly reduced the electric field stimulation-evoked vesicular release in the hippocampus, compared with the control group at S_1_ and S_2_ (control: S_1_: 10.5 ± 1.2 kBq·g^−1^; S_2_: 4.5 ± 0.64 kBq·g^−1^; 0.1 μM: S_1_: 4.4 ± 0.5 kBq·g^−1^; *p* = 0.0002; S_2_: 1.9 ± 0.16 kBq·g^−1^: *p* = 0.0007; 10 μM: S_1_: 1.29 ± 0.24 kBq·g^−1^: *p* < 0.0001; S_2_: 0.83 ± 0.5 kBq·g^−1^: *p* < 0.0001) (Figures 6A,B). The S_1_ and S_2_-induced release of [^3^H] 5-HT was inhibited, indicating that the maximum vesicular axonal activity-induced release was reduced by 87.6%. The tissue’s [^3^H] 5-HT content was decreased in a concentration-dependent manner, as by loading the tissue at 1.0 μM concentration, the storage capacity was inhibited to 91.7 ± 5.84 kBq·g^−1^: *p* > 0.9999, respectively, and by loading the tissue at 10 μM concentration, the storage capacity was significantly altered by 72.4 ± 3.9 kBq·g^−1^: *p* < 0.0001, compared to the control group (381 ± 18.9 kBq·g^−1^) (Figure 6C).

### 3.5 Effects of short-lasting exposure to reserpine, tetrabenazine, and valbenazine on resting and electric field stimulation-evoked vesicular release of [^3^H] 5-HT

In further experiments, to investigate which antagonist binds permanently to the transporters at a concentration of 1 μM, we studied the electric stimulation-evoked vesicular release of radioactivity from hippocampal slices loaded with [^3^H] 5-HT for 45 min. RSP, TBZ, and VBZ (1 μM) were present only during the incubation phase. After 60-min washout periods, RSP significantly reduced the electric field stimulation-evoked vesicular release in the hippocampus throughout the experiment (S_1_: 2.8 ± 0.8 kBq·g^−1^: *p* = 0.0261; S_2_: 1.9 ± 0.4 kBq·g^−1^: *p* = 0.0401), while VBZ increased it (S_1_: 17.1 ± 2.7 kBq·g^−1^: *p* = 0.0335; S_2_: 12.4 ± 1.7 kBq·g^−1^: *p* = 0.0038), compared to the control group (S_1_: 10.1 ± 1.3 kBq·g^−1^; S_2_: 6.3 ± 1.2 kBq·g^−1^). TBZ failed to change the vesicular release (Figures 7A,B). The tissue’s [^3^H] 5-HT content was reduced in the hippocampus by loading the tissue with RSP to 102.0 ± 23.1 kBq·g^−1^: *p* = 0.0009, respectively, compared to the control group (401.5 ± 52.0 kBq·g^−1^). TBZ and VBZ did not alter the tissue’s [^3^H] 5-HT storage capacity (287.0 ± 47.0; 523.0 ± 55.4 kBq·g^−1^: *p* = 0.3223 and *p* = 0.2725) (Figure 7C).

### 3.6 Effects of reserpine i.p. pretreatment on [^3^H] ACh release and on the tissue’s [^3^H] ACh content from hippocampal slices

VAChT (vesicular ACh transporter) and VMAT-2 are members of the SCL18 family of vesicular transporters, and VAChT shares 40% amino acid identity with VMAT-2; therefore, it seemed interesting to study the effect on the vesicular release of ACh. We measured the [^3^H] ACh release from hippocampal slices, from control, and 24 h before the experiment, pretreated (with 1 mg kg^−1^ RSP i.p.) mice. RSP treatment failed to reduce the electric field stimulation-evoked release of [^3^H] ACh (S_1_: 9.08 ± 1.2 kBq·g^−1^:*p* = 0.9197 and S_2_: 7.8 ± 0.72 kBq·g^−1^:*p* = 0.2525) (Figures 8A,B) and the tissue’s [^3^H] ACh content (251.0 ± 19.7 kBq·g^−1^p = 0.1462) compared to the control group (S_1_: 8.92 ± 1.02 kBq·g^−1^ and S_2_: 6.5 ± 0.83 kBq·g^−1^; tissue [^3^H] ACh content: 214.0 ± 12.9 kBq·g^−1^) (Figure 8C). This observation is consistent with the biochemical studies proving that RSP does not affect this transporter (Arvidsson et al., 1997).

## 4 Discussion

In this study, an attempt has been made using neurochemical methods to verify the mode of action of RSP, a competitive antagonist of VMAT-2, compared to the transporter’s non-competitive antagonists, TBZ and VBZ, on the cytoplasmic (resting) and axonal stimulation-evoked vesicular release of NA and 5-HT from *ex vivo* slice preparations dissected from mouse hippocampus, which is involved in memory and learning processes, as well as in depression. A microperfusion system (Sershen et al., 1997) was used to measure the resting and electric field-stimulation-evoked quantal exocytotic release of [^3^H] NA, [^3^H] 5-HT, and [^3^H] ACh. While the resting release of transmitters is [Ca^2+^]_o_-independent and can be increased from the cytoplasm in exchange for extracellular substrates, such as transmitters, by reversing plasma membrane transporter activity, the stimulation-evoked release is [Ca^2+^]_o_-dependent and occurs due to the exocytosis of vesicles in response to action potentials.

As far as the effects on the resting release of amino acid transmitters, using an exchange process was first shown by Levi and Raiteri (1974). The plasma membrane transporter removes the transmitter from the extracellular space, terminating its action. In the case of monoamines, cocaine and antidepressants (SSRI) act by inhibiting plasma membrane transport, thereby increasing the concentration of transmitter in the extracellular space. Regarding resting release, it was first demonstrated that transmitters can enhance cytoplasmic release through an exchange process. Similarly, extraneuronal substrates of the plasma membrane transporter can increase cytoplasmic release of monoamines (Raiteri et al., 1974; Raiteri et al., 1979; Raiteri et al., 2002; Raiteri and Raiteri, 2015; Zsilla et al., 2018). Transporter-mediated, non-vesicular release of DA has even been implicated in modulating social behavior (Roman et al., 2021) and locomotor activity (Tod et al., 2024). These findings support the concept that neuronal communication is not only “digital”—via action potential-triggered vesicular release—but also “analog,” through continuous, graded, transporter-mediated non-vesicular release. The transport activity of VMAT-2 is driven by the proton electrochemical gradient generated by H^+^-ATPase, using two cytoplasmic protons to exchange one cytosolic monoamine (Johnson et al., 1981). The effect of RSP on monoamine levels is accounted for an inhibition of the VMAT-2 located in the membrane of the synaptic vesicles present in the cytoplasm of the brain monoamine nerve terminals. Conditions such as ischemia, hypoxia and hypoglycemia (Milusheva et al., 1996) lead to the loss of ATP production needed to maintain vesicles able to store transmitters and consequently to the inhibition of all the energy-dependent processes, including maintenance of various types of vesicular transporter (Chaudhry et al., 2008) and increasing Ca^2+^-independent release of transmitters from the cytoplasm due to transporter reversal (Milusheva et al., 1996; Zsilla et al., 2018; Lakatos et al., 2020).

In this study, the first neurochemical evidence was obtained that RSP and TBZ/VBZ failed to change resting release but reduced the electric stimulation-evoked exocytotic release of [^3^H] NA and [^3^H] 5-HT and the tissue’s [^3^H] NA (Figures 3–5) and [^3^H] 5-HT uptake (Figures 6, 7) from the hippocampal noradrenergic and serotonergic nerve terminals. The effect of RSP proved to be dose-dependent (Figure 6) and, in contrast to TBZ (Tod et al., 2024), is persistent (Figure 7). Both TBZ and its metabolite, VBZ, exhibit a similar action: reduced axonal activity-dependent exocytotic release of NA. However, under our experimental conditions VBZ transiently increased stimulation-evoked 5-HT release, while TBZ reduced it. This divergent effect can be explained by basic pharmacological properties: TBZ binds more strongly to VMAT-2 and more consistently blocks vesicular uptake, leading to reduced release. By contrast, VBZ is more lipophilic and has distinct pharmacokinetic properties, which allow partial vesicle refilling after washout. This may present as an increase in stimulation-evoked release in our *ex vivo* system. Both drugs have been applied to treat patients suffering from hyperkinetic syndromes (Caroff et al., 2018), occurring as a side effect during the treatment of schizophrenia and major depression with second- (Carbon et al., 2017) and third-generation antipsychotics, including cariprazine and aripiprazole.

Our data support previous biochemical evidence that RSP binds irreversibly to the cytosolic binding site of VMAT-2 (Scherman and Henry, 1984; Wu et al., 2024), thereby preventing vesicular refilling for extended periods, whereas TBZ and VBZ act reversibly, allowing recovery of transmitter content within 24 h. This is consistent with our earlier findings obtained with TBZ in prefrontal cortex preparation (Tod et al., 2024). In the present study RSP failed to increase the stimulation-evoked release of [^3^H] NA in the hippocampus, indicating some refilling of vesicles without influencing the NA transporter (NET). A similar conclusion was drawn with RSP’s effect on the dopamine transporter (DAT) (Metzger et al., 2002). These findings contradict several observations concluding that RSP inhibits reuptake through plasmalemmal monoamine transporters (Ross and Kelder, 1979; Bowyer et al., 1984). One possible explanation for the different interpretation reached in studies using synaptosomal NA content measurement is that inhibition of vesicular monoamine uptake, i.e., the reduced vesicular content due to VMAT-2 inhibition, may have led to a false conclusion by several that it is an effect on plasmalemmal dopamine transporter function. From a translational perspective, this prolonged depletion of vesicular NA and 5-HT in the hippocampus could have behavioral consequences, given the region’s role in mood, cognition, and memory (Vizi and Kiss, 1998), even though in this study no behavioral assays were performed to confirm this link. The observed absence of RSP’s impact on ACh release supports the specificity of its action for VMAT-2 over VAChT (Arvidsson et al., 1997; Eiden et al., 2004). This selectivity is crucial to exclude non-specific vesicular disruption as a mechanism of monoamine depletion.

There has been a long-standing controversy surrounding the theory that RSP treatment may contribute to depressive symptoms by producing its monoamine-depleting effects (Carlsson, 1965). This hypothesis has recently been challenged by a systematic review (Strawbridge et al., 2023), which analyzed clinical and preclinical studies on the effects of RSP. The authors found no consistent evidence that RSP reliably induces depression in humans. Instead, the available data were mixed, with some studies reporting depressive symptoms, while others found no association. These results call into question the long-standing assumption that RSP-induced monoamine depletion directly causes depression. In a longitudinal prospective study, the occurrence of depressed behavior was also studied in patients treated with TBZ (Dorsey et al., 2013). It was concluded that there is no increased risk of depression. The validity of RSP as an animal model for depression remains debated, as some previous studies suggest its action may be transient and relatively mild (Miguel Telega et al., 2024), while others have found that repeated low-dose RSP treatment induces depressive-like behaviors in mice. They showed increased immobility in forced swim and tail suspension tests (Qian et al., 2023). Regarding TBZ behavioral assays have shown that it induces anergia-like states in mice, which were reversed by bupropion, while other characteristics of depression, such as anxiety, sociability, or sucrose consumption, were unaffected by TBZ, and bupropion had no reversing effect on these variables. Furthermore, in touchscreen-based operant tasks, TBZ caused an effort-related motivational dysfunction in mice (Yang et al., 2020), supporting its relevance for motivation models in depression. Since our main goal in this work was to examine presynaptic monoamine release processes in an *ex vivo* setting, we have decided not to conduct behavioral testing. Although this type of preparation enables pharmacological control and high temporal resolution, it is not immediately compatible with behavioral tests. While noting that future research should incorporate behavioral and neurochemical goals for a more comprehensive understanding, we focused on making the mechanism clear because of the study’s aims and resources. Regarding our neurochemical data obtained from mouse hippocampi, the difference between the effects of RSP and TBZ/VBZ on NA release is significant (Figures 3, 5). While RSP in biochemical experiments acts competitively and irreversibly inhibiting the cytosol-facing VMAT-2 (Scherman and Henry, 1984; Wu et al., 2024), in our study, it prevented the refilling of vesicles with transmitters, and its action remained persistent. By contrast, the inhibitory effect of TBZ/VBZ after its removal was short-acting (Figure 7), an observation consistent with biochemical observation and conclusion (Scherman and Henry, 1984) that it is a non-competitive and reversible antagonist, indicating that its effect is surmountable (Wu et al., 2024). In our experiments with RSP, we never observed a recovery in the vesicular release 1 h or a day after its administration (Figures 4, 5), as was the case with TBZ and VBZ (Figure 4). RSP exerts its action persistently (Figures 4, 5), keeping the brain content of monoamine transmitters very low, as it is highly lipophilic and crosses rapidly the blood–brain-barrier (BBB) (Sreemantula et al., 2004), in contrast to TBZ/VBZ, whose action is short-lived, with a rapid recovery to the control level and is surmountable. TBZ rapidly crosses the BBB but undergoes extensive first-pass metabolism to active dihydrotetrabenazine isomers with short half-lives (Schneider et al., 2021; Skor et al., 2017), consistent with the rapid recovery of transmitter release observed in our study. VBZ demonstrates a slower onset but a longer duration of action due to its metabolism and extended plasma half-life (Grigoriadis et al., 2017), which is also reflected in our release measurements.

The more persistent monoamine depletion associated with RSP may contribute to the higher incidence of depressive symptoms reported in some studies, whereas depression during TBZ treatment has been reported less frequently (Dorsey et al., 2013). The treatment of TD with TBZ in doses ranging from 40 to 75 mg·kg^−1^, administered one to three times daily, or VBZ applied once daily at 80 mg·day^−1^ fails to produce intolerable side effects (Caroff et al., 2018). The clinically meaningful improvements in TD without depressive symptoms may be attributed to the very short-lasting VMAT-2 inhibition and some time required for refilling.

## 5 Limitations

There are several limitations to our methods and interpretations. First, only male mice were used, which limits the generalizability of our findings. Sex hormones are known to influence VMAT-2 expression and monoamine signaling, and female rodents may respond differently to VMAT-2 inhibitors (Dluzen et al., 2008). Including both sexes in future work will be essential to capture potential sex-dependent effects.

Second, no behavioral assays were performed in parallel with the neurochemical experiments. Our *ex vivo* microperfusion system allowed high temporal resolution and precise pharmacological manipulation, but it would be necessary to combine neurochemical and behavioral endpoints to get a more comprehensive link between presynaptic mechanisms and functional outcomes.

A potential methodological limitation concerns the possible residual effect of isoflurane used for anesthesia. In our experiments, isoflurane was applied only briefly (≥1.5 v/v%) to induce rapid loss of consciousness, followed immediately by decapitation. The brain was then immersed in ice-cold, oxygenated Krebs buffer, and hippocampal slices were washed three times before the uptake phase and superfused for 60 min prior to stimulation. During this period, the incubation and perfusion media were continuously replaced, allowing effective washout of any volatile anesthetic residues. Isoflurane is a volatile agent with a relatively low blood/gas partition coefficient (1.45 ± 0.12), enabling rapid equilibration and elimination from the brain (Esper et al., 2015). *In vivo* ^19^F-NMR studies have shown that isoflurane is cleared from the brain with a fast half-life of approximately 7–9 min, followed by a slower redistribution phase (Chen et al., 1992). Given these pharmacokinetic properties and our extended washing and perfusion protocol, any residual isoflurane in the slices is expected to be negligible. Moreover, all control and drug-treated groups underwent identical anesthesia and tissue preparation procedures; therefore, any group differences in stimulation-evoked transmitter release can be attributed to the pharmacological actions of RSP, TBZ or VBZ, rather than anesthetic carry-over. Consistent with this, stimulation-evoked [^3^H] monoamine release in control slices was robust, tetrodotoxin-sensitive, and within physiological ranges, arguing against tonic suppression of transmitter release by residual anesthetic. Nevertheless, a minor transient modulatory effect of isoflurane on monoaminergic transmission cannot be completely excluded and should be considered as a potential limitation.

## 6 Conclusion

In summary, we provide neurochemical evidence that RSP, as a competitive, and TBZ and VBZ, as non-competitive, antagonists of VMAT-2, concentration- and dose-dependently inhibit the amount of [^3^H] NA and [^3^H] 5-HT released in response to axonal stimulation without affecting the activity of monoamine plasmalemmal transporters from the hippocampus and the storage capacity. Furthermore, RSP fails to affect the uptake and the exocytotic release of [^3^H] ACh from *ex vivo* hippocampal slices.

## Data Availability

The original contributions presented in the study are included in the article/supplementary material, further inquiries can be directed to the corresponding author.
